# How are the employed and unemployed affected by the economic crisis in Spain? Educational inequalities, life conditions and mental health in a context of high unemployment

**DOI:** 10.1186/s12889-016-2934-z

**Published:** 2016-03-15

**Authors:** Juan Antonio Córdoba-Doña, Antonio Escolar-Pujolar, Miguel San Sebastián, Per E. Gustafsson

**Affiliations:** Delegación Territorial de Igualdad, Salud y Políticas Sociales de Andalucía, Avda María Auxiliadora 2, 11009 Cádiz, Spain; Department of Public Health and Clinical Medicine, Epidemiology and Global Health, Umeå University, SE-901 85 Umeå, Sweden; Department of Public Health and Clinical Medicine, Social Medicine, Umeå University, SE-901 85 Umeå, Sweden

**Keywords:** Economic crisis, Mental health, Employment status, Educational inequalities, Financial strain, Social support, Spain

## Abstract

**Background:**

Despite an increasing number of studies on the factors mediating the impact of the economic recession on mental health, research beyond the individual employment status is scarce. Our objectives were to investigate in which ways the mental health of employed and unemployed populations is differently affected by the current economic recession along the educational scale and to examine whether financial strain and social support explain these effects of the crisis.

**Methods:**

A repeated cross-sectional study, using two waves of the Andalusian Health Survey in 2007 (pre-crisis) and 2011–2012 (crisis). A population aged between 19 and 64 years was selected. The dependent variable was the Mental Component Summary of the SF-12 questionnaire. We performed Poisson regression models stratified by working status, with period, educational level, financial strain and social support as independent variables. We examined interactions between period and educational level. Age, sex, main earner, cohabitation and partner's working status were considered as covariates.

**Results:**

The study included 3210 individuals (1185 women) in 2007 and 3633 individuals (1486 women) in 2011–2012. In working individuals the prevalence of poor mental health increased for secondary and complete primary studies groups during crisis compared to the pre-crisis period, while it decreased significantly in the university study group (PR = 0.76, 95 % CI: 0.58–0.99). However, in unemployed individuals prevalence ratios for poor mental health increased significantly only in the secondary studies group (PR = 1.73, 95 % CI: 1.06–2.83). Financial strain and social support yielded consistent associations with mental health in all subgroups. Only financial strain could partly explain the crisis effect on mental health among the unemployed.

**Conclusions:**

Our study supports the finding that current economic recession is associated with poorer mental health differentially according to labour market status and educational level. Those with secondary studies may be at risk in times of economic recession. In connection with this, emerging educational inequalities in mental health among the employed population were observed. Our research also suggests a partial mediating role of financial strain for the effects of crisis on poor mental health among the unemployed. Good social support appears to buffer poor mental health in all subgroups but not specifically during crisis period.

**Electronic supplementary material:**

The online version of this article (doi:10.1186/s12889-016-2934-z) contains supplementary material, which is available to authorized users.

## Background

There is growing evidence of the deleterious effects on mental health of the current recession which started in 2008 [1]. This body of research has investigated a diversity of outcome measures such as suicide, suicidal attempts, suicide ideation, mental disorders or perceived mental health, among others [2]. A considerable number of studies has been carried out in Southern Europe where the impact of the so called Great Recession on national and local economies was more detrimental [3]. In the case of Spain, the crisis hit the country’s economy hard, leaving in its wake a substantial rise in poverty and social inequalities [4], as well as impairments in the health care system and population health indicators [5].

Recent research has also ventured beyond the simple mental health effects of the crisis, in attempts to understand not only if, but also how and for whom, the crisis impacts on population mental health. For example, in examinations of plausible individual mediating factors for the association between economic recession and mental health, most research has focused on the role of unemployment [6]. Acknowledging the causal relationship between labour market status and psychological well-being [7], much attention has thus been paid to the mental health problems of the unemployed men and women during the crisis period [8]. However, very scarce efforts have been devoted to the investigation of mental health effects of economic crises on active workers, despite research having revealed that unemployment does not explain all the changes in population mental health due to crisis [9, 10]. One example is a recent study in Korea reporting that as many as half of the suicides during a deep economic crisis occurred in the employed population [11], and recent evidence indicates that research that goes beyond the employment-unemployment axis is required to understand mental health in times of economic recessions [12]. Thus, it is important to understand the potential mediating role of variables other than unemployment, such as socioeconomic position and social support.

Along this line of thought, there is a growing acceptance of the fact that economic crises are complex events that affect health-related behavioural patterns via various and even opposing mechanisms and pathways [13]. However, when alternative mediators such as income, educational attainment or social support have eventually been considered in reports on economic recessions, they have been studied separately [14]. Therefore, little is known about their joint or independent roles in explaining how economic recession impacts on mental health.

Another question with regard to the impact of the economic recession is how it affects particularly vulnerable population groups and the social distribution of mental health. However, even though research highlights that in times of economic stability mental disorders more frequently affect the unemployed population, people in the lower income brackets, in lower educated groups or with less social support [15], only quite recently has particular attention been paid to social inequalities in mental health during an economic recession [16].

With the purpose of disentangling how the recession may affect mental health, we carried out our study in Andalusia, the southernmost region of Spain which has a very high structural unemployment rate. As our indicator of inequality we first considered educational level, which is accepted to be quite stable over the life cycle and so a very useful socioeconomic position variable in most settings [17]. As potential mediators we selected financial strain, to approach the acute economic situation of the person and his or her family, and social support, as its potential buffering effect against the negative effects of the recession on mental health has recently been addressed [18], and research on a previous crisis in Spain has noted the relevance of the mechanisms of familial solidarity to protect its members from the fluctuations of economic and employment cycles [19].

The general aim of the present study was to investigate in which ways the mental health of the employed and unemployed were affected by the current economic crisis. The specific aims were to examine, among the employed and unemployed:

1) whether mental health changed differently from before to after the crisis along the educational level scale (aim 1.a), thus influencing educational inequalities in mental health (aim 1.b), and

2) whether individual material (aim 2.a) and social (aim 2.b) conditions explained mental health and educational inequalities in mental health before and after the crisis.

## Methods

### Setting

**Fig. 1:**
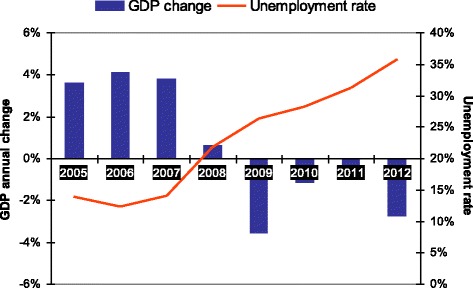
GDP annual change and unemployment rate (both sexes) in Andalusia from 2005 to 2012

Our study was carried out in Andalusia, the most populated region in Spain, with around 8.5 million inhabitants. The majority of its social and economic indicators are below the Spanish average, though Andalusia has considerably overcome its previous economic lag in the last decades. For instance, per capita GDP was 16960€ in 2012, 25.5 % lower than the Spanish average. Unemployment rose in Andalusia from 12.2 % in 2006 to 35.8 % in 2012 (from 8.3 to 25.8 % in Spain) [20], and poverty rates increased from 29.5 % in 2008 to 31.0 % in 2012, above the Spanish poverty rate of 22.2 % [21]. Trends in GDP annual change and unemployment rate are provided in Fig. 1.

The Spanish health system is essentially decentralised, and each one of the 17 regions has a high level of autonomy. Health coverage in Andalusia, including emergencies and mental health services, is provided on a universal coverage basis. The total service provision in primary health care and emergencies is publicly managed, and only 5 % of publicly funded hospital services are privately provided. Until 2015 there were no user fees, and co-payment was required only at ambulatory pharmacies (with exemptions for the elderly and the unemployed). Mental health care in Andalusia is mainly provided by the public health system.

### Sample

For the present study, we chose a repeated cross-sectional design, using two waves of the Andalusian Health Survey [22]: 2007 for the pre-crisis period and 2011 (February 2011 to February 2012) for the crisis period. Data were accessed with permission of the Regional Health Authority (Secretaría General de Salud Pública de la Junta de Andalucía). These complete data set is available for researchers upon request to the aforementioned department.

The Andalusian Health Survey has been carried out every 4 years since 1999. It uses a probabilistic multistage cluster and stratified sampling procedure. The survey includes non-institutionalised adults of 16 years and older. A design effect of 1.35 was used in sample size calculations. In 2007 there were 6511 people interviewed and 6507 in 2011–2012. Field substitution was used during the survey process to compensate for non-response. For our study the sample was limited to the population between 19 and 64 years who were employed or unemployed according to self-reported working status at the time of the interview. In this way retired persons, students and men or women devoted to housework as their main occupation were dropped. That yielded 3210 individuals (2025 men and 1185 women) in 2007 and 3633 individuals (2147 men and 1486 women) in 2011–2012. Missing data were negligible for the variables included in the analyses. The study is subject to the Spanish legislation on data protection [23]. All participants gave their informed consent to be included in the study. During analysis questionnaire data were unlinked from any personal identification information to guarantee anonymity. Additionally, as the national law provides, the file containing personal data held by the public authority, in our case the Autonomous Region of Andalusia, was registered in the General Data Protection Registry [24]. Thus, the study, periodically conducted by the Regional Health Authority, did not need further formal ethics approval.

### Variables

We used the Mental Component Score (MCS) of the Short Form Health Survey (SF-12) questionnaire as the outcome variable [25]. This 12-item scale has been adapted and validated for the Spanish population [26] and has been included in all the waves of the Andalusian Health Survey. We used it as a dichotomous variable, categorising the first quintile (20 % with lower score) as poor mental health and the rest as good mental health. This led to a cut-off point of 47.3. There is no universally accepted cut-off level. Ours is intermediate between the previously used of 45 for screening of depressive disorder and 50 for any common mental disorders [27]. Sensitivity analyses instead using a cut-off point of 49.8 (corresponding to the lowest quartile) were also performed, yielding virtually identical results (data not reported).

Employment status, with the above mentioned categories (unemployed and employed) was used as stratification variable, since we were interested in examining the differential impact of crisis on mental health in these two groups. Unemployed included both workers without a current occupation and people in search of a first job. The variable period included two categories, pre-crisis and crisis, corresponding to the two waves of the survey, 2007 and 2011.

As exposures we included two socioeconomic status variables. Educational level was categorised as: no studies or incomplete primary; complete primary; secondary; and university studies. The second socioeconomic status variable was financial strain, measured through a question on difficulties to make ends meet, widely used in social and economic surveys [28]. This variable was originally recorded in six categories and later recoded into three: great difficulty or difficulty to make ends meet; some difficulty; and ease.

Social support was measured by the Duke scale [29]. This questionnaire measures both confidant and affective support. It includes 11 items on a 5-point scale ranging from 1 to 5, which are summed up into an index, with higher total score indicating higher level of social support. This variable was dichotomised using a cut-off point at percentile 15 of the total score, proposed for the Spanish population [30]. This led to a threshold of equal or greater than 41 for good social support.

Other covariates included were sex; age in years, with five categories: 19–24, 25–34, 35–44, 45–54 and 55–64; main earner, as a dichotomous variable (yes/no); cohabitation (yes/no); and partner’s working status considered as: unemployed, working and other status.

### Analysis

We calculated the crude prevalence of poor mental health in all the categories of the variables in each period, stratifying by employment status. Chi-square tests were performed to assess mental health differences between periods in all the categories separately for employed and unemployed.

We estimated adjusted prevalence ratios (PR) of poor mental health using Poisson regression models. In order to address the general aim of examining the effects of crisis among employed and unemployed, all main analyses were also stratified by employment status and reported separately.

In Model 1 we included the period variable and education, as well as sex, age, main earner condition, cohabitation and partner working status as adjustment variables. To address aim 1, an education*period interaction term was added in Model 2 and in the following models. Results in Models 2, 3 and 4 are reported in two complementary ways. First, according to aim 1.a., PRs corresponding to the exponentiated linear combinations of the main effects coefficients and interaction coefficients were estimated, with the period effect (PRs; precrisis vs crisis) reported within each educational category. Here, the reference is the prevalence of each educational category in the precrisis period. Second, corresponding to aim 1.b., the educational level effects (PRs) are reported within each period. Here, the university studies educational group within each period (precrisis and crisis) is the reference category. Finally, to address the second aim, financial strain (Model 3; aim 2.a) and social support (Model 4; aim 2.b) were added.

In the final models we also calculated the Relative Index of Inequality (RII) for educational level and poor mental health, to summarize the magnitude of educational inequalities within each period in one estimate. RII is a measure of relative health inequalities, based on the ranking of the socioeconomic variable, and is easily calculated by different regression methods. In our case, it can be interpreted as the effect on mental health of moving from the lowest to the highest educational level group.

In order to explore whether results were different for women and men, analyses were also rerun separately for each sex. Since estimates were similar in both sexes (data not provided), final analyses were only done on collapsed data to achieve more robust estimations, with sex only included as a covariate.

Analyses were performed with Stata software version 13. The STROBE statement was used to ensure the reporting of this observational study. See Additional file 1.

## Results

The characteristics of the sample by working status and period are shown in Table 1. The majority of the variables remained considerably stable across periods. The proportion of unemployed in the sample was 13.7 % in 2007 and 39.3 % in the crisis period. As expected, there were important differences between periods regarding partner's working status and difficulty to make ends meet distributions.

**Table 1:** Characteristics of the sample

	Employed		Unemployed	
Variables	2007	2011	2007	2011
	*n* = 2771	*n* = 2205	*n* = 439	*n* = 1428
	%	%	%	%
Mental Health				
Poor (MCS-SF12 score <47.3)	17.1	19.0	22.6	26.1
Good (MCS-SF12 score > =47.3)	82.9	81.0	77.4	73.9
Sex				
Women	35.0	39.6	49.2	42.9
Men	65.0	60.4	50.8	57.1
Age				
19–24 years	10.1	7.0	19.1	13.8
25–34 years	30.8	28.6	40.5	29.3
35–44 years	30.2	31.1	19.4	28.6
45–54 years	20.6	23.7	13.0	19.0
55–64 years	8.3	9.6	8.0	9.3
Main earner				
Yes	62.6	62.0	26.4	40.5
No	37.4	38.0	73.6	59.5
Cohabitation				
Yes	70.5	72.9	45.6	63.1
No	29.5	27.1	54.4	36.9
Partner working status				
Employed	57.8	59.1	62.5	50.0
Unemployed	4.1	12.4	12.5	28.1
Other	32.1	28.5	22.0	21.9
Education				
No studies or Incomplete primary	17.5	13.6	29.8	25.5
Complete primary	27.8	26.0	34.9	31.9
Secondary	30.7	37.6	21.4	31.2
University	24.0	22.8	13.9	11.4
Difficulties to make ends meet				
Great difficulty/Difficulty	15.5	21.3	36.8	52.2
Some difficulty	31.9	27.6	37.7	30.5
Easily	52.6	51.1	25.5	17.3
Social support				
Poor (Duke score < =41)	19.6	17.5	17.3	12.4
Good (Duke score >41)	80.4	82.5	82.7	87.6

The prevalence of poor mental health in each category of the independent variables stratified by working status and period is presented in Table 2. Overall crude prevalence of poor mental health increased in the second period in both working status strata (from 17.1 to 19.0 in employed and from 22.6 to 26.1 in unemployed), though differences were not statistically significant.

**Table 2:** Prevalence of poor mental health (low mental component score SF-12) by employment status and period

	Working			Unemployed		
	Precrisis	Crisis	*p*	Precrisis	Crisis	*p*
	*n* = 2771	*n* = 2205		*n* = 439	*n* = 1428	
	%	%		%	%	
All	17.1	19.0		22.6	26.1	
Sex						
Women	22.1	23.9		25.9	29.6	
Men	14.4	15.8		19.3	23.5	
Age						
19–24 years	18.6	12.3		16.7	20.3	
25–34 years	14.5	15.9		16.8	22.9	
35–44 years	19.1	21.8		32.9	29.7	
45–54 years	17.3	20.5		28.1	29.1	
55–64 years	16.5	20.4		31.4	29.3	
Main Earner						
Yes	15.1	17.8		27.6	27.1	
No	20.4	21.0		20.7	25.4	
Cohabitation						
Yes	16.4	18.8		22.0	25.3	
No	18.6	19.3		23.0	27.3	
Partner working status						
Employed	19.5	19.1		21.4	22.3	
Unemployed	12.5	19.5	*	20.0	29.9	
Other	12.2	18.1	*	25.0	26.8	
Education						
No studies or Incomplete primary	21.0	24.7		28.2	26.9	
Complete primary	16.5	21.6	*	27.4	24.5	
Secondary	14.6	18.6	*	14.9	30.5	*
University	18.0	13.3	*	9.8	17.2	
Difficulties to make ends meet						
Great difficulty/Difficulty	22.9	32.4	*	35.0	31.9	
Some difficulty	16.6	17.9		13.6	21.8	*
Easily	15.6	14.0		17.4	16.3	
Social support						
Poor (Duke score < =41)	32.2	36.1		45.3	42.4	
Good (Duke score >41)	13.9	16.6	*	17.0	22.7	*

*P* p-value of the χ^2^ test for the comparison of prevalence of poor mental health precrisis versus crisis for each category. *: *p* < 0.05

Among the employed, the prevalence of poor mental health increased during the crisis period in all educational groups, except for the university group where it decreased (from 18.0 to 13.3, *p* < 0.05). Differently, among the unemployed population only in the secondary studies group a significant increase in poor mental health was detected.

### Estimating the mental health effect of crisis on employment status and educational subgroups

The results of the Poisson regression models are presented in Tables 3 (employed) and 4 (unemployed). In the initial Model 1, we found a non-significant 9–10 % increase in the adjusted prevalence of poor mental health both in employed and unemployed strata. Education was independently associated with poor mental health in both employment strata.

**Table 3:** Poisson regression models for prevalence ratios of poor mental health in employed Andalusian population

	Model 1		Model 2		Model 3		Model 4	
	PR	CI95	PR	CI95	PR	CI95	PR	CI95
Period								
Crisis/Precrisis	1.09	0.96–1.23						
Education								
University	ref							
Secondary	1.08	0.91–1.28						
Primary complete	1.22	1.03–1.46						
Primary incomplete	1.45	1.20–1.76						
Period - Education								
Crisis/Precrisis – University			0.73	0.56–0.97	0.74	0.56–0.97	0.76	0.58–0–99
Crisis/Precrisis – Secondary			1.24	1.00–1.54	1.20	0.97–1.49	1.23	1.00–1.52
Crisis/Precrisis – Primary complete			1.25	1.00–1.56	1.19	0.95–1.48	1.27	1.02–1.57
Crisis/Precrisis – Primary incomplete			1.09	0.84–1.42	1.07	0.82–1.39	1.16	0.89–1.50
Education – Precrisis								
University			ref		ref		ref	
Secondary			0.86	0.68–1.08	0.80	0.63–1.01	0.79	0.62–0.99
Primary complete			0.98	0.78–1.30	0.89	0.70–1.12	0.82	0.65–1.03
Primary incomplete			1.24	0.97–1.58	1.07	0.84–1.38	0.99	0.78–1.26
Education – Crisis								
University			ref		ref		ref	
Secondary			1.44	1.11–1.88	1.30	1.00–1.70	1.28	0.98–1.67
Primary complete			1.66	1.27–2.18	1.43	1.08–1.88	1.37	1.04–1.81
Primary incomplete			1.84	1.36–2.49	1.56	1.15–2.11	1.51	1.12–2.05
Difficulty to Make Ends Meet								
No difficulty					ref		ref	
Difficulty					1.16	1.00–1.35	1.12	0.97–1.30
Great difficulty					1.75	1.51–2.03	1.64	1.41–1.90
Social Support								
Good social support/Low social support							0.49	0.43–0.56

Models adjusted for sex, age, main earner condition, cohabitation and partner's working status

*PR* prevalence ratio, *CI95* 95 % confidence interval

**Table 4:** Poisson regression models for prevalence ratios of poor mental health in unemployed Andalusian population

	Model 1		Model 2		Model 3		Model 4	
	PR	CI95	PR	CI95	PR	CI95	PR	CI95
Period								
Crisis/Precrisis	1.10	0.91–1.34						
Education								
University	ref							
Secondary	1.82	1.30–2.55						
Primary complete	1.55	1.11–2.18						
Primary incomplete	1.62	1.14–2.29						
Period - Education								
Period – University			1.64	0.72–3.76	1.46	0.64–3.36	1.40	0.61–3.19
Period – Secondary			2.09	1.27–3.44	1.85	1.11–3.06	1.73	1.06–2.83
Period – Primary complete			0.79	0.59–1.07	0.74	0.55–1.00	0.78	0.59–1.05
Period – Primary incomplete			0.96	0.70–1.33	0.94	0.68–1.28	0.98	0.72–1.33
Education – Precrisis								
University			ref		ref		ref	
Secondary			1.43	0.58–3.51	1.28	0.52–3.19	1.25	0.51–3.05
Primary complete			2.72	1.22–6.07	2.24	1.00–5.03	1.96	0.88–4.38
Primary incomplete			2.43	1.08–5.48	1.89	0.84–4.28	1.63	0.72–3.70
Education – Crisis								
University			ref		ref		ref	
Secondary			1.82	1.27–2.60	1.62	1.13–2.32	1.54	1.08–2.19
Primary complete			1.31	0.90–1.91	1.14	0.79–1.66	1.10	0.76–1.58
Primary incomplete			1.43	0.97–2.09	1.21	0.83–1.76	1.14	0.78–1.65
Difficulty to Make Ends Meet								
No difficulty					ref		ref	
Difficulty					1.23	0.92–1.64	1.21	0.91–1.61
Great difficulty					1.90	1.46–2.48	1.78	1.37–2.33
Social Support								
Good/Low							0.57	0.49–0.68

Models adjusted for sex, age, main earner condition, cohabitation and partner's working status

*PR* prevalence ratio, *CI95* 95 % Confidence Interval

In Model 2 (adding education*period interaction term) we detected a differential effect of crisis by educational level in both employed and unemployed. Specifically, in working people, we detected that the prevalence of poor mental health decreased significantly during crisis in the university study group compared to the pre-crisis period (PR = 0.73, 95 % CI: 0.5–0.97), while it tended to increase in the other three lower education groups, significantly for secondary (PR = 1.24, 95 % CI: 1.00–1.54) and complete primary studies (PR = 1.25, 95 % CI: 1.00–1.56). In the unemployed people (Table 4), we observed a different distribution of the period effect across educational groups: poor mental health increased from pre-crisis to crisis periods in the secondary studies group (PR = 2.09, 95 % CI: 1.27–3.44) with a similar but non-significant pattern for the university group. The effect, although not significant, changed insubstantially or in the opposite direction in the two lower educational level groups.

Concerning educational inequalities in mental health effects, an educational gradient was shown in mental health among employed during but not before crisis. The PR between lowest educated and highest educated became significant in the second period (PR = 1.84, 95 % CI: 1.36–2.49), as well as the PR between workers with complete primary studies (PR = 1.66, 95 % CI: 1.27–2.18) and secondary studies (PR = 1.44, 95 % CI: 1.11–1.88). Correspondingly, the RII increased numerically from a non-significant 0.97 (95 % CI: 0.76–1.43) in the precrisis period to a significant 1.57 (95 % CI: 1.13–2.17) in the crisis period. Among unemployed, from the educational inequality perspective, in the pre-crisis period the two lower educated groups presented poorer mental health compared to the university studies group, but during crisis only the secondary studies group presented a significantly higher PR than the reference group (PR = 1.82, 95 % CI: 1.27–2.60). The RII was not significant in either period, changing from 1.56 (95 % CI: 0.83–2.94) in the precrisis to 0.76 (95 % CI: 0.55–1.06) in the crisis period.

### Assessing the role of financial strain and social support

Both financial strain and social support were strongly and independently associated with poor mental health consistently in employed and unemployed. Nevertheless, the inclusion of these two variables in the models (Models 3 and 4) only reduced the PR for poor mental health in the unemployed, decreasing the effect size of crisis by 15 % in the university and secondary studies subgroups. For example, for secondary studies PR was attenuated to 1.73 (95 % CI: 1.06–2.83). The main proportion of this attenuation (around 12 %) could be attributed to financial strain (Model 3). No attenuation in the coefficients for any educational subgroup was observed in the employed population.

From the educational inequalities perspective a slight attenuation of PRs was also observed in the unemployed group.

## Discussion

This study suggests a differential effect of the economic crisis on mental health according to employment status and educational level. On the one hand, among the employed, the crisis seems to worsen mental health only in the intermediate educational groups, while working people with university studies improved their self-reported mental health during the recession compared with the pre-crisis period. On the other hand, among the unemployed we observed a divergent distribution of the period effect across educational groups: crisis was associated with a negative effect on mental health in people with secondary studies, with no association detected in the highest educated and in the two less educated categories. A second interesting finding is that educational inequalities in poor mental health emerged from before to during the economic recession in the employed population, while rather a reverse pattern of decreasing educational inequalities was seen among the unemployed. Our results further indicate that financial strain may be partially mediating the effect of crisis on mental health in the unemployed, and that social support is strongly associated with mental health in all population subgroups but is not a mediator of the crisis effect.

The first question this study sought to determine was whether mental health changed differently from before to after the crisis along the educational level scale in employed and unemployed. One possible interpretation of our results is that among working people, only those with higher educational attainment feel secure in times of crisis, while those in the lower education strata are more affected by job insecurity. Our results are thus consistent with those reported by Lam et al., who performed a study in the USA excluding self-employed workers, comparing two moments, 2006 and 2010, and reported a middle-class vulnerability during the economic downturn. They argue that elevated unemployment rates occurring in crises may exacerbate the effect of job insecurity on mental health mainly in the third quartile of personal income, which they consider middle class [31].

Worse mental health in the crisis period among the unemployed secondary studies and complete primary studies groups could indicate that these educational groups are not prepared for the new and rapidly established economic context, in contrast to the lowest educated group, for which unemployment and long term unemployment has historically been more prevalent in this region. For example, individuals with higher education may have more positive expectations about their chances of finding a satisfactory job, thus easing their anxiety during unemployment [32]. It has further been argued that the unemployed with university studies can consider working abroad or other possibilities. Indeed, some estimations indicate that more than half a million Spaniards have migrated to another European country since the beginning of the recession; most of them are young, and a great proportion have a university degree [33].

Surprisingly, we also found that the second educational level group was doubly affected by the crisis, not only when unemployed but when employed too. These results are in agreement with a study in Iceland that detected that adults in middle-income families suffered increased stress levels after the economic downturn [34]. The authors argue that several factors such as decreasing purchasing power and a higher proportion of homeowners defaulting on their mortgages could play a role. Similarly to what happened in Spain and Andalusia, important inversions in high-priced real estate during the housing boom might have hit middle-income families most severely, thus resulting in economic insecurity eventually leading to increased stress levels.

From the inequality perspective, we detected an educational gradient in mental health among the employed during the economic recession which was not observed in the period of economic stability. To the authors’ knowledge, this finding has not been reported in previous research, which as mentioned above has instead been mainly set on the deteriorating effect of increasing unemployment [8]. On the other hand, among the unemployed, the stronger specific effect of crisis on the secondary studies group and to a lesser degree on the university group switched the observed educational gradient detected in the pre-crisis period. A recent study performed in Spain detected an increase in educational mental health inequalities in men but not in women comparing the 2006 and 2011 national health surveys [6]. These results are partially in agreement with our research, though further comparisons of changes in mental health during the crisis period could not be done as data were not stratified by employment status.

With regard to potential material and social mediators (aim 2), the results show that a great difficulty to make ends meet is significantly associated with poor mental health in both groups (adjusting for educational level and the rest of the variables). Only in unemployed did we detect a possible mediation role of the financial strain in the effect of crisis on mental health. We think these findings are expected, as current lack of economic resources may influence mental health status [35, 36]. Economic difficulties play a role on mental health status independent from educational level both in workers and unemployed. In this study it seems that it works independently from working status.

We also detected a very consistent and strong association between social support and mental health, in both working status groups. This association has already been described as independent from employment status, though not specifically during an economic downturn [37]. The addition of the variable social support in the regression models only produced minor changes in the effect of crisis on poor mental health in the diverse educational groups, thus we cannot consider it as a mediator but a variable operating both during and between crisis periods. Thus, social support and mainly family support could help to explain the apparent contradiction between high levels of unemployment and low indicators of social distress in comparison with other countries where unemployment is lower not only during recession periods. There is evidence from the current recession that informal networks in Spain continue to perform as buffers against the risks of the labour market and lack of confidence in the state welfare policies [38, 39]. Although our study was conducted in the Andalusian population, our main results may be generalized to the Spanish population, based on cultural similarities and comparable social and economic impact of current recession.

### Limitations

Our study presents the limitations of a repeated cross-sectional design. We used two waves of a survey four years apart and thus were unable to measure the variation in the intensity of the crisis between the two time points. Because of this limitation, the associations detected should be interpreted with caution.

Another potential source of uncertainty could be the representativeness of our sample. To assess this point we analysed the distribution of key variables in the sample and in the population. For instance the proportion of unemployed in the sample was 39.3 % in the crisis period. This finding correlated with the official unemployment statistics that reported a rate of 35.9 % unemployment in the region by the end of 2012.

We have used subjective indicators of financial distress, so we cannot discard that it is possible that individuals changed their reference point when their assessment of difficulty to make ends meet was performed the during crisis period. It is unfortunate that this study was limited by the lack of information on individual unemployment protection [40]. This variable is not included in the Andalusian Health Survey. Another uncontrolled factor is the absence of data regarding job precariousness or part-time working. Both situations, related to mental health [41], are increasingly prevalent in Spain after new labour market regulations implemented during the present austerity period.

## Conclusions and policy implications

The present study aimed to determine the effect of the current economic recession on mental health in the employed and unemployed population in a high structural unemployment region. The evidence of this study suggests that the current economic recession is associated with poorer mental health differentially according to labour market status and educational level. Specifically, those with secondary studies present more vulnerability for poorer mental health in times of economic recession compared to previous periods. Moreover, the crisis saw an emergence of educational inequalities in mental health among the employed population, but rather decreasing inequalities among the unemployed. Our research also detected a partial mediating role of financial strain in the effects of crisis on poor mental health among the unemployed. Good social support appears to buffer poor mental health in all subgroups in our context, but not specifically during a crisis period. Taken together, the present study suggests that the effect of economic crisis on mental health is influenced by complex interactions between employment status and socioeconomic variables that must be considered to target appropriate interventions.

Our findings reinforce the importance of developing prevention initiatives intended to reach the specific populations more exposed to the adverse effects of economic recessions [11]. Beyond the necessary overall strategies like effective labour market programmes and reinforcement of current social networks, the identification of more vulnerable subgroups such as secondary studies, employed and unemployed, poses a challenge for social services and social safety nets [1], more frequently oriented to lower socioeconomic brackets.

The combination of findings of this study also has implications for primary health care and mental health services. Access to quality primary and mental health services are essential in reducing the individual risks evidenced in the current recession [35]. However, though universal health care coverage in Spain has been guaranteed so far for the vast majority, it has been reported to be in jeopardy due to austerity policies affecting financing cuts and staff reductions.

## Additional file

Additional file 1: STROBE Statement—Checklist of items that should be included in reports of cross-sectional studies. (DOC 60 kb)
